# Recent Progress in Functionalized Coatings for Corrosion Protection of Magnesium Alloys—A Review

**DOI:** 10.3390/ma15113912

**Published:** 2022-05-31

**Authors:** Bingzhi Li, Zhaoqi Zhang, Tengteng Liu, Zhenghui Qiu, Yan Su, Jinwei Zhang, Cunguo Lin, Li Wang

**Affiliations:** 1 State Key Laboratory for Marine Corrosion and Protection, Luoyang Ship Material Research Institute, Qingdao 266101, China; lbz11666888@163.com (B.L.); zhangzhaoqi527@163.com (Z.Z.); liutt@sunrui.net (T.L.); qiuzh@sunrui.net (Z.Q.); zhangjw@sunrui.net (J.Z.); 2 Southwest Technology and Engineering Research Institute, Chongqing 400039, China; suyan71@126.com

**Keywords:** magnesium alloys, corrosion resistance, superhydrophobic coatings, self-healing coatings, functional coatings

## Abstract

Magnesium (Mg) and its alloys, which have good mechanical properties and damping capacities, are considered as potential candidate materials in the industrial field. Nevertheless, fast corrosion is the main obstacle that seriously hinders its wide applications. Surface modification is an available method to avoid the contact between corrosive media and Mg substrates, thus extending the service life of Mg-based materials. Generally, manufacturing a dense and stable coating as physical barriers can effectively inhibit the corrosion of Mg substrates; however, in some complex service environments, physical barrier coating only may not satisfy the long-term service of Mg alloys. In this case, it is very important to endow the coating with suitable functional characteristics, such as superhydrophobic and self-healing properties. In this review, the various surface treatments reported are presented first, followed by the methods employed for developing superhydrophobic surfaces with micro/nanostructuring, and an overview of the various advanced self-healing coatings, devolved on Mg alloys in the past decade, is further summarized. The corresponding preparation strategies and protection mechanisms of functional coatings are further discussed. A potential research direction is also briefly proposed to help guide functional strategies and inspire further innovations. It is hoped that the summary of this paper will be helpful to the surface modification of Mg alloys and promote the further development of this emerging research field.

## 1. Introduction

Magnesium (Mg) alloy, as the lightest structural metal in practical application, is widely used in aerospace, electronics, and automobile fields because of its excellent characteristics [1], such as low density, high specific strength, as well as good electrical and thermal conductivity [2,3]. With the increasingly serious global resource crisis, light metal materials represented by Mg alloys are highly favored. However, the standard electrode potential of Mg is −2.37 V/SCE [4], indicating a strong tendency of corrosion [5]. Moreover, when compared to Ti and Al alloys having compact passive surface film, a less corrosion-resistant film, consisting of internal Mg-O and external porous Mg(OH)_2_ layers, is generated on the Mg surface [6]. The low anti-corrosive performance of Mg substrates often leads to the destruction of their mechanical integrity in the early stage, which seriously restricts their broad applications in the engineering field [7]. Therefore, appropriate strategies are necessary to slow down the corrosion rate of Mg alloys.

Some researchers have put forward several strategies to ameliorate the anti-corrosion performance of Mg-based materials [8], and several reviews are available where more details of Mg corrosion and its protection strategies can be referred [9,10]. Among those different corrosion protection strategies, alloying design, as one of the effective methods, refers to adding elements to pure Mg so that the metal Mg can become an alloy with expected properties [11,12]. Studies have shown that the element of iron (Fe), aluminum (Al), manganese (Mn), copper (Cu), zinc (Zn), silicon (Si), calcium (Ca), yttrium (Y), zirconium (Zr), and rare-earth elements can affect the corrosion resistance and mechanical properties of Mg [13,14]. The anti-corrosive capability of Mg alloys can be significantly affected by adjusting the types and contents of added alloy elements, especially in the control of the second phase. Nevertheless, there are some shortcomings; for instance, too many alloy phases can lead to a galvanic reaction of Mg alloy, resulting in the decreased corrosion resistance of Mg substrates [15,16].

Surface treatment, as another alternative tactic, has been widely employed to restrain the corrosion behavior of metallic Mg materials without changing the substrate properties [17]. Numerous surface modification methods, including chemical conversion film, electrodeposition, micro-arc oxidation, electrospinning, as well as chemical etching [17,18,19,20,21,22], can endow the Mg material with functional characteristics such as superhydrophobic and self-healing properties, which can further ameliorate the anti-corrosive ability of Mg alloys. At present, a mass of literature has reviewed the advances in the cutting-edge development of coatings for Mg alloys. However, those reported reviews focus, primarily, on the preparation strategy of Mg alloys for industrial applications, but they focus rarely on the functional characteristic of coatings for Mg materials; under severe corrosion conditions, the conventional surface-modified coatings often have defects, such as local corrosion, which leads to the failure of protection. Moreover, most of the damaged coatings need cumbersome repair or replacement; otherwise, it will lead to premature failure of the coating. The coating with superhydrophobic function can pass through the air layer between micro and nano, effectively avoiding the enrichment of the corrosive medium in local areas and preventing local corrosion. The coating with self-healing function can actively heal when defects occur, thus avoiding cumbersome replacement procedures. Therefore, developing a functional coating with self-healing or superhydrophobic properties on the Mg alloy is very important.

**Figure 1:**
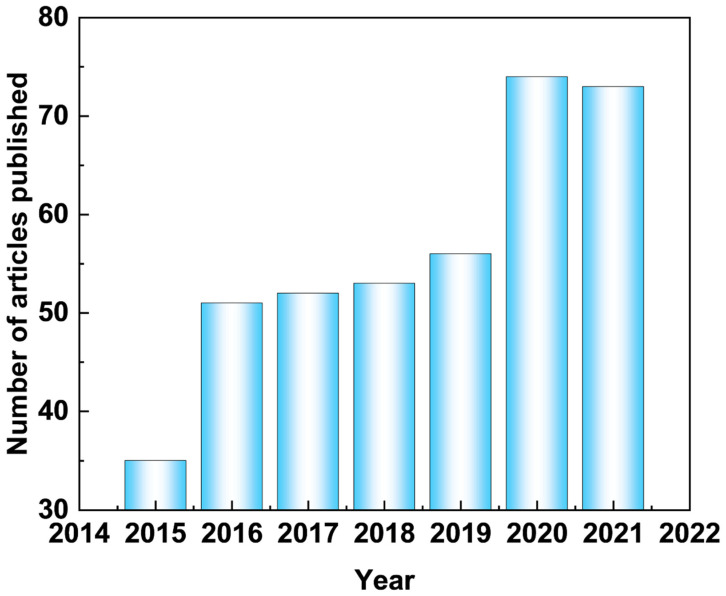
Statistical publications indexed in the web of science by using the term “Superhydrophobic” and “Magnesium alloy”. The number of publications from 2015 to 2021.

**Figure 2:**
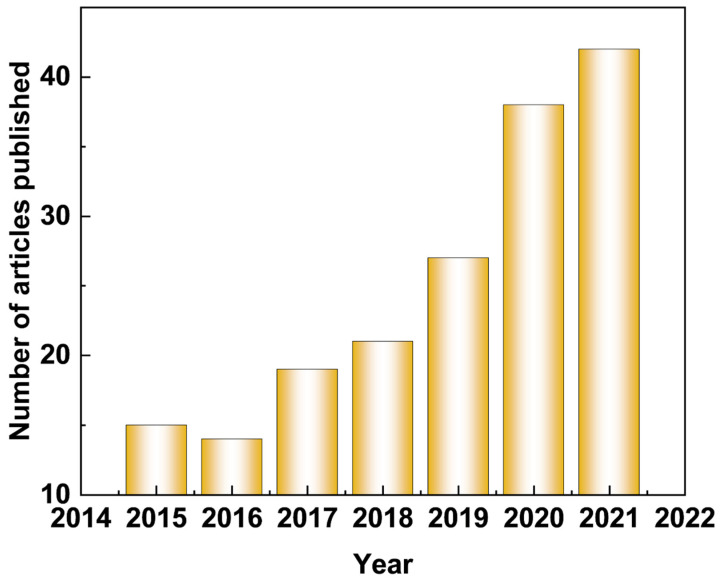
Statistical publications indexed in the web of science by using the term “Self-healing/repairing” and “Magnesium alloy”. The number of publications from 2015 to 2021.

By merely searching the term “Superhydrophobic” and “Magnesium alloy” in the web of science, until 2021, more than 500 records are found, which indicates an accelerated increase in the number of publications (Figure 1). Figure 2 depicts the published paper of self-healing coating on Mg alloys retrieved from the Web of Science. A dramatically increasing number of published papers can be obtained in recent years, indicating that an increasing number of researchers have focused on this area. This review is a retrospective on the latest developments of functionalized coatings of Mg and its alloys to improve the corrosion resistance, including superhydrophobic and self-healing protective coatings. Besides, the preparation methods, characteristics, challenges, and development directions of functionalized coatings for Mg alloys are also discussed and proposed. It is hoped that this review could help researchers understand the development of functional coatings in a timely manner, thereby promoting the industrial application of Mg alloys.

## 2. Superhydrophobic Coatings

**Figure 3:**
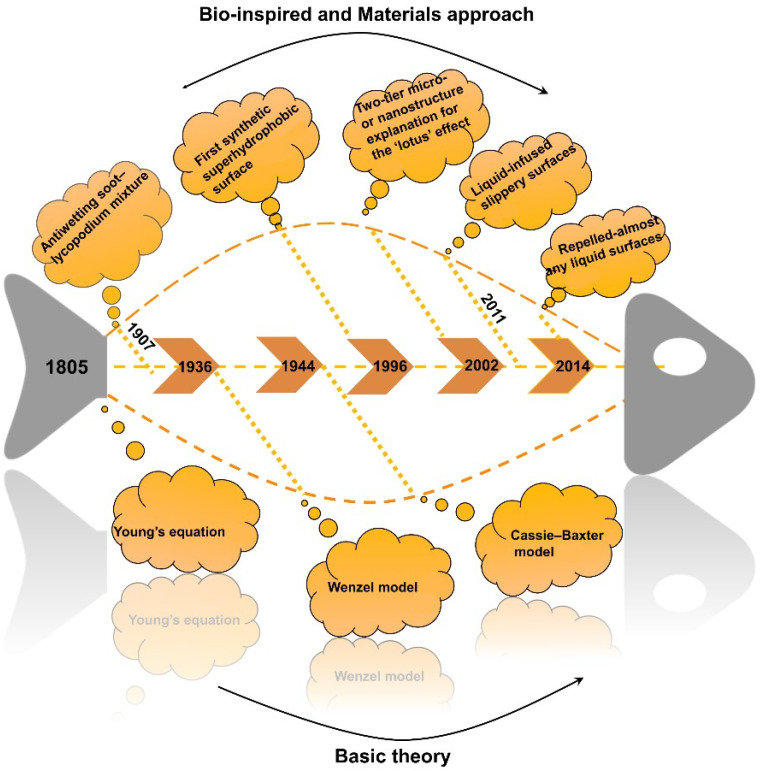
Chart of the major discoveries and developments in the field of superhydrophobic coatings [23,24,25,26,27,28,30,31].

The research on superhydrophobic behavior goes back a long time, as shown in Figure 3. Thomas Young, a pioneer of scientific research on wetting, established Young’s equation in 1805 to propose the concept of the contact angle of a liquid and to define the notion of surface wettability [23]. In 1907, Ollivier observed that superhydrophobic phenomena appeared on surfaces coated with soot, arsenic trioxide, and lycopodium powder [24]. Afterward, in 1923, Coghill and Anderson found that, after the deposition of stearic acids on the surface, galena showed a high superhydrophobic feature [24]. Soon after, Wenzel developed a relationship between the macro roughness and the contact angle of a solid surface and explained how roughness can enhance hydrophobicity in 1936 [25]. Later, Cassie and Baxter (in 1944) found porous surfaces and rough surfaces that can trap air between water and a solid [26]. However, due to the limitation of technological development, the research on superhydrophobic surfaces was relatively limited. Until the introduction of the electron microscope in the early 1970s and botanist William Bartlett’s exploration of the lotus leaf phenomenon, the superhydrophobic surface gradually attracted people’s attention. The first synthetic superhydrophobic surface was manufactured in 1996 by introducing roughness on alkyl ketene dimer film using a bottom-up strategy [27]. In 2002, inspired by lotus and rice leaves, Feng’s research showed that micro/nano-scale double-layer structures have been proven to be significant players in realizing the high apparent contact angle and low adhesion of lotus leaves [28]. Liu et al. [29] investigated, in 2009, that the wetting/anti-wetting behavior of liquid droplets on a solid surface is not an apparent or simple contact between two phases but among three phases. To further improve the stability of the superhydrophobic surface, inspired by the Nepenthes pitcher plant, Wong et al. [30] reported synthetic liquid-repellent surfaces in 2011. Up to now, no surface can repel liquids with extremely low-energy, such as fluorinated solvents. In 2014, Liu and Kim used the concept of an umbrella to make a kind of material whose surface can repel almost any liquid [31].

The process of wetting a solid surface with water is of great significance in surface science, and hydrophobic, as well as hydrophilic, surfaces receive a prominent role in industrial applications. Among them, the static contact angle (SCA) > 150° and sliding angle (SA) < 10° are defined as superhydrophobic surfaces [32]. For the contact state of liquid on a solid surface, Thomas Young, a pioneer scientist who worked in 1805, proposed the concept of the contact angle of a liquid and developed Young’s equation to define the notion of surface wettability:

(1) $$cos\theta_{Y}=\left(\sigma_{SA}-\sigma_{SL}\right)/\sigma_{LA}$$

where $\;\theta_{Y\;}$ indicates intrinsic contact angle or Young’s contact angle and $\sigma_{SA}$, $\sigma_{SL}$, and $\sigma_{LA}$ means interface energy between solid-gas, solid-liquid, and liquid-gas interface. To facilitate better observations of the contact angle at asperity surfaces. Young’s equation was revised by Wenzel et al. (1936). When the three-phase contact line on the rough surface moves *dx* outwards, the change of interface energy *dF* is:

(2) $$dF=r_{R}\left(\sigma_{SL}-\sigma_{SA}\right)dx+\sigma_{LA}dx\cos\theta_{W}$$

where $r_{R}$ indicates roughness factor, and $\theta_{W}$ means the apparent contact angle of liquid droplets on a rough surface. When the interface energy of the system is the smallest, the system is in equilibrium. At this point, $\theta_{W}\;$ can be represented as:

(3) $$\cos\theta_{W}=r_{R}\left(\sigma_{SA}-\sigma_{SL}\right)/\sigma_{LA}=r_{R}\cos\theta_{Y}$$

**Figure 4:**
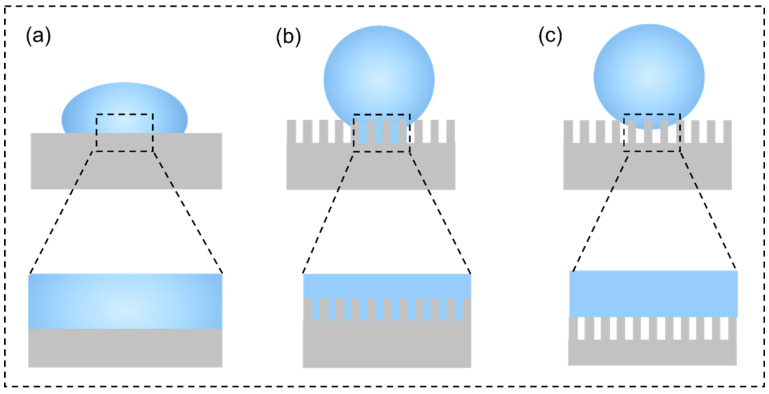
Three theoretical models: (**a**) Young model (**b**) Wenzel model, (**c**) Cassie–Baxter model.

The equation reveals the important influence of roughness on wettability, and it is found that the roughness structure can enhance the wettability of the substrate. In 1944, Cassie and Baxter studied the wetting behavior of porous surfaces, and based on Wensel’s equation, they established the relationship between the apparent contact angle and the inherent contact angle in the non-uniform wetting state. The change of interface energy *dF* in Cassie state is:

(4) $$dF=\emptyset_{SL}\left(\sigma_{SL}-\sigma_{SA}\right)dx+\sigma_{LA}\emptyset_{SA}dx+\sigma_{LA}dx\cos\theta_{CB}$$

where $\emptyset_{SL}$ indicates the area fraction between the solid and liquid interface, and $\emptyset_{SA}$ means the area fraction between solid and air interface. Different from Wenzel’s state, the air layer inside the micro-nano structure, in the Cassie–Baxter state, cannot be wetted by water, and the adhesion to water droplets is smaller. The three theoretical models are shown in Figure 4.

**Figure 5:**
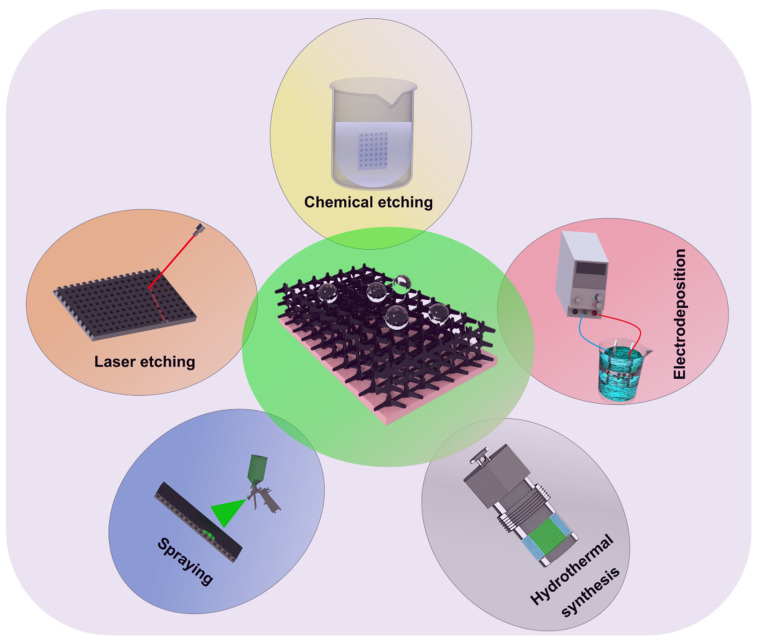
Fabrication strategies of superhydrophobic coatings.

For a hydrophilic surface, the corrosive solution is easy to diffuse to Mg materials and lead to corrosion. In contrast, superhydrophobic coatings, composed of low surface energy substances and rough micro-nano structures [33], demonstrate significant corrosion protection for Mg substrate, and the corrosion protection mechanism can be attributed to a “three barriers” corrosion protection [34]. The first barrier is the existence of air between the corrosive medium and the surface, which can reduce the corrosion area; the subsequent barrier layer is the low surface energy layer, which can effectively inhibit the electric transfer in the corrosion process due to poor conductivity; this is followed by a dense rough layer, which prevents the corrosion solution from penetrating. Hence, numerous fabrication strategies are established to construct the superhydrophobic coating and for improving the corrosion resistance of Mg alloys, including micro-arc oxidation (MAO), chemical etching, hydrothermal synthesis, electrodeposition, spraying, and sol-gel methods (Figure 5) [32,34].

### 2.1. Micro Arc Oxidation

Micro arc oxidation (MAO) refers to a novel technology for in-situ growth of ceramic oxide film on an Mg alloy, and the formed MAO coating shows a rough microstructure and is firmed to Mg substrate [35,36,37]. MAO can significantly improve the corrosion resistance of Mg alloy substrates. However, the porous morphology, produced in the preparation process, is not conducive to the corrosion prevention of Mg alloys. It is found that post-treatment can make up for the defects of porous morphology and significantly improve the corrosion resistance of the Mg alloy matrix. Guo et al. [38] sealed MAO porous structure through physical interlocking and prepared MAO/PLLA composite coating to improve the corrosion resistance of Mg alloys. To further enhance the corrosion resistance of Mg alloys, Liu et al. [39] found that chitosan (CHI) and poly (styrene sulfonate) (PSS) polyelectrolyte multilayers were fabricated on the MAO-treated Mg alloy via the layer-by-layer (LBL) self-assembly technique to close the porous flaws. In recent years, the use of superhydrophobic coatings and other post-treatment to make up for porous defects has attracted great attention. The first superhydrophobic coating on Mg alloys was reported by Liang et al. in 2007 [40]. This team applied the MAO as the pretreatment and then soaked the MAO sample in acrylic acid to prepare a superhydrophobic surface. Subsequently, more and more researchers are beginning to pay attention to this field [41]. Cui et al. [42] found that a superhydrophobic surface could be created on an MAO-modified Mg alloy AZ31 by stearic acid (SA) treatment, and the SA/MAO treated AZ31 Mg alloy achieved a maximum CA of 151.5° after 10 h of modification. The immersion test indicated that, compared to the AZ31 substrate and MAO coating, the superhydrophobic MAO-SA coating availably protected the Mg substrate against corrosion for at least 11 d in 3.5 wt.% NaCl solution, demonstrating that superhydrophobic surfaces offered better long-range corrosion guard for AZ31 Mg alloys. However, unwanted side reactions would occur during the aforementioned superhydrophobic coating preparation process. Based on the above problem, the unique interactions between hexadecanoic acid (HA) and albumin (ALB) molecules, on the surface of the porous layer of AZ31 Mg alloys, were exploited by Kaseem et al. to fabricate a novel hybrid composite film with excellent anti-corrosion in a 3.5 wt.% NaCl solution [43]. In addition, we can also refer to other literature [42,44,45,46,47,48] on improving the corrosion resistance of Mg alloys by post-treatment. To further improve the adhesion of modified molecules with low surface energy, some literature reports that boiling the initial prepared MAO coating increases the surface density of chemisorption-active sites: that is, −OH group [49]. However, although MAO coating has good corrosion resistance, and it is also suitable to be modified into the superhydrophobic surface, the processing area of a single sample is limited due to the need for high voltage and high current density, which limits the wide application of the MAO method in the engineering field.

### 2.2. Chemical Etching

Without expensive equipment, special conditions, or complex procedures, the chemical etching method is a good candidate to produce superhydrophobic coatings. Chemical etching is to construct the rough structure on the Mg surface through the redox reaction of metal oxidant and the metal itself, and then, the superhydrophobic coating can be obtained by the modification of low surface energy substances [50]. Wan’s team [51] proposed a quick acid etching strategy to produce a surface with a micro/nanostructure, which only took about 5 min, and the SA was used to embellish the etched surface. Results suggested that the prepared coating had a low CA of 142° and showed good corrosion resistance. To improve the hydrophobic performance of the developed coating, Wei’s group [52] synthesized a superhydrophobic layer on the AZ31 and AZ91 alloy sheets through simple etching and SA modification. Results demonstrated that the CA of the coating was as high as 160° when etching for 50 s, confirming a superior superhydrophobicity of this as-fabricated surface. The coating also showed good corrosion resistance in 3.5 wt.% NaCl solution, which was due to the presence of an air layer in the dense multi-scaled structure of the superhydrophobic surface.

### 2.3. Hydrothermal Synthesis

**Figure 6:**
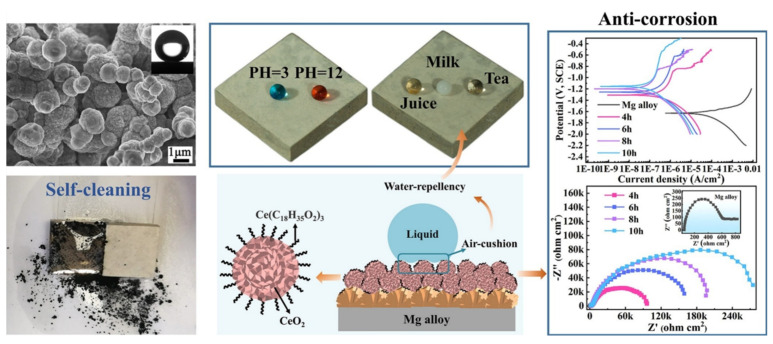
The schematic diagram for the fabrication process of superhydrophobic coatings on the Mg alloy. Reprinted with permission from Ref. [57]. Copyright (2021) Elsevier.

Hydrothermal treatment is a strategy in which the precursor is placed in an autoclave and reacted at high pressure and temperature, and this method is widely applied to develop superhydrophobic films on Mg alloys because of its simple process, low cost, high yield, and the easy control of its crystal shape [53,54]. Wang’s team [55] applied the hydrothermal means to fabricate a lamellar magnesium hydroxide (Mg(OH)_2_) layer on the AZ91 alloy, followed by the modification of SA. The as-prepared superhydrophobic coating had a CA of 155° and an SA of ~2°. The optical images proved that the superhydrophobic film could provide long-term corrosion protection for the Mg alloy substrate. Similarly, layered double hydroxide (LDH) coating, with the ability to anti-corrode of Cl^−^ ions, prepared by hydrothermal treatment, was applied to create a superhydrophobic coating on Mg alloys by SA treatment and was reported by Zhou et al. [56]. Results indicated this developed Zn-Al LDH-SA coating exhibited a high CA of 165.6°, and the CA remained larger than 150° when exposed to the air for 100 d, revealing favorable durability. To avoid a complicated preparation process, Chu et al. [57] developed a convenient one-step hydrothermal method to form a superhydrophobic coating composed of CeO_2_ and cerium stearate (Ce(CH_3_(CH_2_)_16_COO)_3_) (Figure 6). The coating, with an allium giganteum-like structure, presented a micro-nano hierarchical roughness, and it had a high CA of 164° and a low SA of 4.1°. Moreover, the superhydrophobic coating remarkably enhanced corrosion resistance arises from the decrease in the contact area between the corrosion solution and sample due to the water-repellency of the coating. Unfortunately, the hydrothermal method is not suitable for large-area preparation of coatings.

### 2.4. Electrodeposition

**Figure 7:**
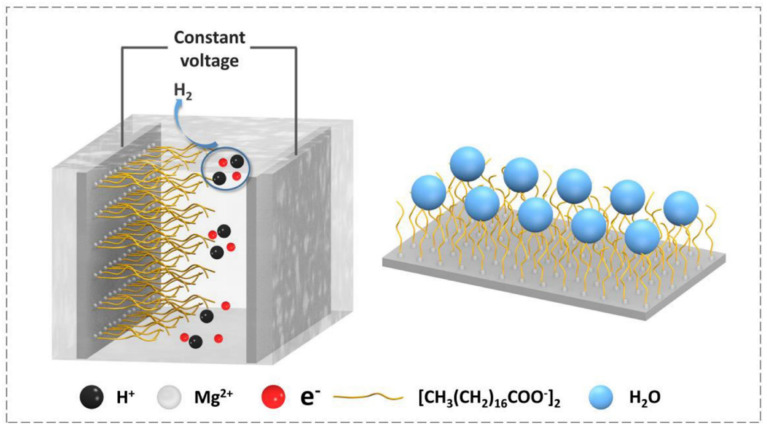
Scheme of the one-step electrodeposition on Mg alloys. Reprinted with permission from Ref. [7]. Copyright (2019) Elsevier.

Electrodeposition is an alternative method to fabricate nano-micro structures on Mg alloys [58]. The process is simple and easy to control, so the cost is low, and the preparation efficiency is high, and batch preparation can be realized [7,59,60,61,62]. At present, researchers focus on Ni-based layers on Mg alloys by the electrodeposition method, followed by modifying the surface with low surface energy [63]. Liu et al. [63] prepared cauliflower-like superhydrophobic coatings (a CA of 160.8 ± 1° and an SA of 1.8 ± 1°) on AZ91D Mg alloys by Ni electrodeposition and SA modification, which was employed to reform the anti-corrosive ability of Mg alloys. In the 3.5 wt.% NaCl solution, electrochemical impedance spectroscopy (EIS) showed that the impedance modulus |Z| of superhydrophobic coatings was larger than that of the bare substrate, implying that the coating had good corrosion resistance. To improve deposition efficiency and reduce cost, Pan et al. [7] proposed a simple method for preparing the superhydrophobic magnesium stearate coating on the AZ31 Mg alloy by one-step electrodeposition, which was because the negatively charged long-chain molecules with low surface energy can easily be electrostatically attracted to the Mg surface by metal cations [34], and this team put the AZ31 Mg alloy in an ethanol solution, containing SA and magnesium nitrate (Mg(NO_3_)_2_), for 30 min and prepared a magnesium stearate (Mg[CH_3_(CH_2_)_16_COO]_2_) coating, with a CA of 156.2° by electrodeposition. Corrosion resistance suggested that the obtained superhydrophobic surface availably protected the Mg substrate for at least 7 d (Figure 7). However, the adhesive force and stability of all electrodeposited superhydrophobic coatings need to be strengthened further.

### 2.5. Spraying

**Figure 8:**
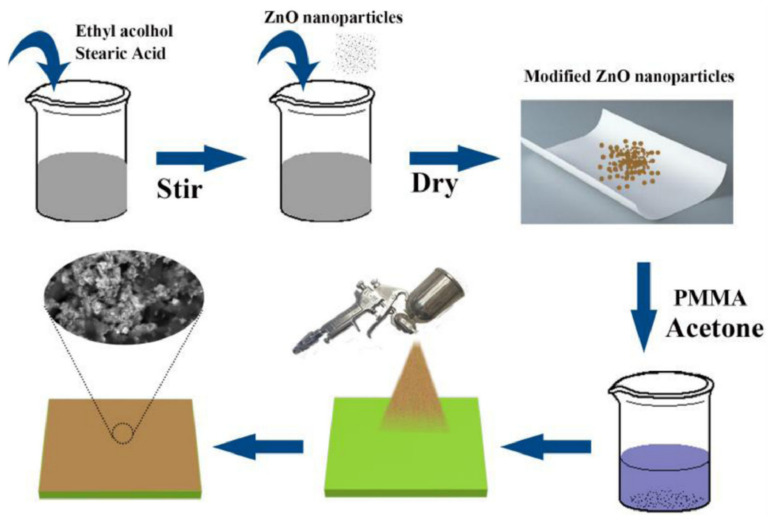
Schematic illustration of the fabricating process. Reprinted with permission from Ref. [66]. Copyright (2021) Elsevier.

Among various reported methods to fabricate a superhydrophobic surface, the spraying method is a promising one because of its easy handling, convenience for large-scale application, environmental friendliness, and simplicity to repair by respraying [64,65]. The spraying method is to prepare the spraying solution first and then spray the solution, by spraying machine, to obtain the rough structure on the surface [66,67]. Shi et al. [68] used the spraying method to prepare a solid superhydrophobic composite coating on AZ31 Mg alloys by spraying the polyphenylene sulfide (PPS), polytetrafluoroethylene (PTFE), and SiO_2_ nanoparticles, and the PPS-PTFE/SiO_2_ coating showed good adhesion to the substrate due to the good adhesion of PPS to the metal substrate. EIS plots confirmed that the superhydrophobic layer provided a good corrosion guard for AZ31 Mg alloys. However, the above superhydrophobic coating always suffers from the poor durability that is caused by the incompatibility of nanoparticles. To resolve these problems, Li et al. [66] developed a simple method, based on spraying, for constructing the superhydrophobic coatings with superior robustness on Mg alloys (Figure 8), and this team sprayed ZnO nanoparticles modified by SA (dissolved in acetone poly (methyl methacrylate) (PMMA)) onto the AZ31 Mg alloy substrate. The coating showed good mechanical stability in various physical and chemical damage experiments, and it also had high anti-corrosion performance.

### 2.6. Sol-Gel

To produce nanostructured superhydrophobic coatings with various morphological characteristics, the sol-gel method is considered to be one of the most preferred methods. Sol-gel technology is a simple industrial process that can be used in a variety of coatings on a broad range of materials, especially in the design of complex shapes [69]. Wang et al. [70] employed the sol-gel strategy to create a superhydrophobic silica coating (a CA of 151º) on the MAO-modified NZ30K Mg alloy, and an electrochemical test indicated that the silica coating significantly improved the corrosion resistance of Mg alloys (the *i*_corr_ decreased by three orders of magnitude) in 3.5 wt.% NaCl solution. Immersion tests (168 h of soaking) confirmed that the superhydrophobic silica film can effectively supply long-term corrosion guards for NZ30K Mg alloys. However, the superhydrophobic silica coating prepared by the sol-gel tactic is time-consuming, and this method has poor film-forming properties, which is attributed to the shrinkage of the film due to the removal of many gases and organics in the drying process [71].

In the previous part, we summarize the preparation strategy and protection mechanism of superhydrophobic coatings to abate the corrosion behavior of Mg alloys (Table 1) [7,33,42,52,57,68,70,72,73,74,75,76,77], which is helpful for researchers to develop superhydrophobic film with enhanced anti-corrosion characteristics on the Mg alloy. The advantages and disadvantages of various preparation technologies were compared. The results show that electrodeposition, spraying, hydrothermal, and chemical etching method have simple equipment, high efficiency, and economy; the electrodeposition and spraying methods, especially, can be used for large-scale preparation in the industrial field. However, there are some shortcomings in the preparation process, such as the poor bonding force between the coating and Mg substrate. Micro-arc oxidation can effectively improve the adhesion, but the preparation process involves high voltage and high current, which is not conducive to the popularization of the industrial field. At this stage, one of the main limitations hindering the use of superhydrophobic coatings in industrial protection applications is their poor durability. Although significant progress has been made in the development of anti-corrosion superhydrophobic coatings, most studies and reports are still limited to the laboratory. It is certain that, due to the simplicity and cost-effectiveness of the strategy, it must become a leading technology for Mg alloy protection in the next few years.

## 3. Self-Healing Coatings

**Figure 9:**
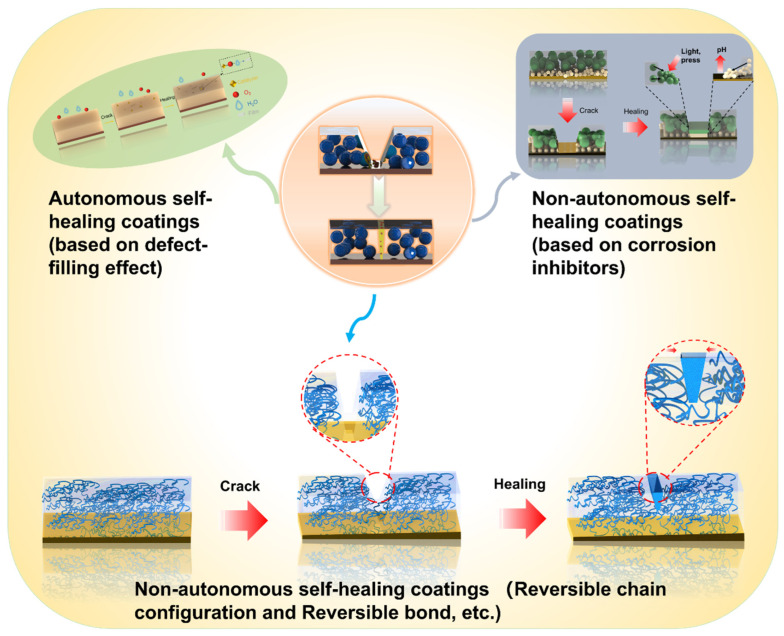
Schematic illustrations of the healing process of self-healing coating.

Generally, the physical barrier coating can meet the requirements of corrosion resistance of Mg alloys, but in some complex service environments, the physical barrier coating will inevitably be damaged during use [78]. At present, most of the damaged coatings need to be repaired or replaced manually, and the cost is high. Researchers found that designing a self-healing coating that changes its performance in response to environmental stimuli has proved to be a possible way to solve this problem [79]. Self-healing coating that is endowed with bionic self-healing function through technical improvement based on ordinary physical barrier coating. When the coating is damaged, the damaged part of the coating can be repaired with minimal or no external intervention. Self-healing ability can enhance the protective capability of coatings and prolong the service life of Mg alloys [80]. Therefore, the development of an advanced protective coating with intelligent self-healing characteristics is very important for the corrosion shield of an Mg substrate [81]. According to whether the healing process occurs automatically, the self-healing coating is divided into autonomous and non-autonomous, as shown in Figure 9 [82].

### 3.1. Autonomous Self-Healing Coatings

**Figure 10:**
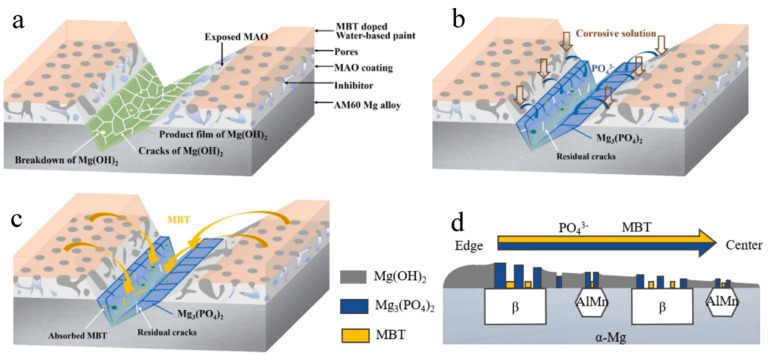
Schematic illustrations of the healing process of self-healing coating. Reprinted with permission from Ref. [83]. Copyright (2019) Elsevier.

Autonomous healing coating refers to the coating, itself, that can repair its bulk integrity or functional properties, without any external physical intervention, when it is damaged, as shown in Figure 10 [83,84]. There are main methods to achieve autonomous healing: (a) the first is chemical conversion, which is a tactic to convert the surface of active materials into passivation. The self-healing performance of chemical conversion coating mainly depends on the dissolution reprecipitation of local defects [85]; (b) the second type is to embed a polymerizable healing agent in the coating. In many cases, these healing agents are stored in microcapsules. When the coating is damaged, the capsule ruptures and releases the healing agent, and then, it polymerizes to form a protective film for repairing the physical barrier of the coating [86]; (c) the other kind of self-healing protective coating uses corrosion inhibitor as a repair agent, which can penetrate the coating defects and impede the electrochemical reaction on the exposed metals [83,87].

Early investigation and application of self-healing coatings mostly focused on the chemical conversion film. At present, the most commonly used conversion films include phosphate conversion film, chromate conversion film, cerium salt, and so on [88,89]. Guo et al. [90] developed a Ce-V conversion coating by immersing the AZ31 Mg alloy into the conversion solution composed of 19.7 mM NaVO_3_ and 0.92–46.1 mM Ce(NO_3_)_3_·6H_2_O at 50 °C for 20 min. Results showed that the additions of Ce(NO_3_)_3_·6H_2_O prevented pentavalent V from reducing to tetravalent V in the coating during the conversion reaction process. Adding an appropriate amount of Ce(NO_3_)_3_·6H_2_O to the V solution can obtain a thicker coating with a more compact CeVO_4_ layer. Electrochemical tests indicated that the Ce-V coating can improve the corrosion resistance, which was attributed to the self-healing ability of the Ce-V coating provided by the release and migration of V compounds. The preparation method of chemical conversion film is simple, and the cost is low, but the corrosion resistance is limited due to the poor compactness, easy cracking, and low adhesion of the coating [91].

Self-healing coating can also be achieved by embedding corrosion inhibitors in the coatings, and corrosion inhibitors are divided into inorganic inhibitors (i.e., phosphate, nitrite) and organic inhibitors (i.e., benzotriazole (BTA), mercaptobenzothiazole (MBT), 8-hydroxyquinoline (8-HQ)) [82]. Gnedenkov et al. [92] applied the MAO technology (electrolyte includes 15 g of Na_2_SiO_3_·5H_2_O and 5 g of NaF) to create a ceramic magnesia (MgO) coating on the MA8 alloy. Then, the MAO-treated alloy was immersed in the 8-HQ solution for 120 min, followed by drying for 10 min. Scanning vibration electrode technology (SVET) suggested that the coating led to the decrease in the *i*_coor_ in 30 fold (from 100 μA cm^−2^ down to 3.2 μA cm^−2^) in the corrosion conditions (0.05 M NaCl) and prevented the intensive destruction of materials. However, the above coatings have problems of poor adhesion and an uneven layer of coating. To get around this problem, Adsul et al. [93] used naturally available clay nanotubes (halloysite) loaded with cationic corrosion inhibitors Ce^3+^/Zr^4+^ to disperse in the organic-inorganic sol-gel matrix; subsequently, a novel self-healing coating on the AZ91D alloys can be prepared by the dip-coating method (cured at 130 °C for 1 h in the air). This outcome showed the hybrid sol-gel film, loaded with corrosion inhibitor, showed more promising corrosion resistance when compared to just the sol-gel matrix coating, after prolonged exposure to corrosive environments.

### 3.2. Non-Autonomous Self-Healing Coatings

**Figure 11:**
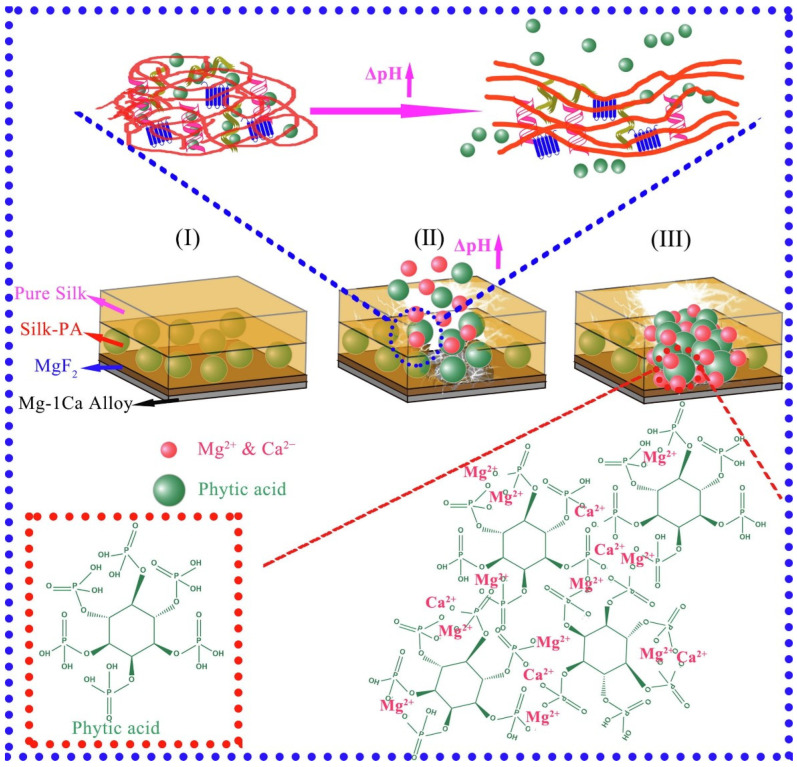
Schematic of the self-healing mechanism upon silk-PA exposure in the immersion environment. Reprinted with permission from Ref. [94]. Copyright (2019) Elsevier.

Non-autonomous self-healing coating means that, when the coating is damaged, it cannot repair the damage automatically, and external stimuli (light, heat, pH, etc.) are required to achieve the healing function. Xiong et al. [94] designed a sandwich-like, pH-responsive, and self-healing silk-phytic acid coating system on the Mg-1Ca alloy. This coating system can be divided into three parts with respective functions: the bottom layer of fluoride conversion film, the middle layer of silk-phytic acid (PA) coating, and the top layer of silk fibroin coating. The silk-PA enhanced the corrosion-resisting capability of bare Mg-1Ca alloy, even after 14 d of exposure to the corrosive media. Scratch and SVET tests further substantiated the self-healing functionality of the silk-PA layer (Figure 11). Wu et al. [95] designed a photo-controlled self-healing coating of polypyrrole (PPy)/polycaprolactone (PCL) hybrid coating on MgSr alloys. The photothermal effects of PPy, inspired by near-infrared light (NIR) irradiation (808 nm), can locally heat the coating to the low melting-point temperature required for PCL within 6 min. The scratched region can get the desired recovery of its corrosion resistance after NIR irradiation, as a result of the synergistic function of low-melting-point deformation of the coating matrix and the photothermal effect of PPy. However, although the non-autonomous self-healing coating can quickly repair the damaged coating, this process needs external stimulation, which has some limitations.

In the previous part, we provided a comprehensive review of the autonomous and non-autonomous self-healing mechanisms harnessed for extending the lifetimes of coatings for corrosion protection. One of the most straightforward methods for the fabrication of autonomous healing coating is to embed healing agents in the coating. In many cases, these healing agents are stored in microcapsules. When the coating is broken, no external special stimulation is required, and the capsules rupture in response to the mechanical impacts and release the healing agents. The self-healing coating can quickly respond to the corrosion damaged area of the coating and quickly repair the damaged area through the release of a corrosion inhibitor. However, there is a defect that the self-healing coating loses its self-healing function after all the corrosion inhibitors are released. Non-autonomous self-healing can remedy the flaws of losing self-healing function. The main advantage of non-autonomous self-healing coating is that, theoretically, the repair process can be repeated for an infinite number of times without requiring any second phase healing agents. However, non-autonomous self-healing coating needs to be repaired under special stimulation (light, temperature), which limits its application.

## 4. Comparison and Challenges in the Functional Coating on Mg Alloys

In summary, functional coatings (superhydrophobic and self-healing coatings) can significantly improve the corrosion resistance of Mg alloys and have important application prospects in the industrial field. Table 2 shows a summary of the corrosion studies of functional coatings on Mg alloys [7,42,54,56,57,59,63,72,96,97,98]. Among them, the anti-corrosion efficiency of coatings is obtained via the following formula:

(5) $$\;\eta =\frac{I_{corr}^{0}-I_{corr}}{I_{corr}^{0}}$$

The inhibition efficiency (*η*) is used to evaluate the corrosion protection performance of the coating. *I*^0^_corr_ and *I*_corr_ are the corrosion current densities obtained through the potentiodynamic polarization test in 3.5 wt.% NaCl solutions.

It can be found that superhydrophobic and self-healing coatings can efficaciously reduce the *i*_corr_ of the Mg substrate (usually by three orders of magnitude), and the anti-corrosion efficiency remains over 99% in most coatings, revealing that Mg alloys are efficiently protected. In order to evaluate the durability of superhydrophobic and self-healing coatings, we evaluated the durability by counting the long-term immersion time of coatings in different papers. Unfortunately, most papers did not conduct long-term immersion experiments. Therefore, we presented the data of immersion time in Table 2. Comparing the above data, this result indicates that the soaking time of most superhydrophobic coatings was 24–250 h. It can be found that the microstructure and low surface material of superhydrophobic coatings are easy to damage or strip, showing poor durability, which reveals that the poor durability of superhydrophobic coatings may not provide long-term protection [99,100,101]. The self-healing coating, as another functional coating, are an efficacious tactic to ameliorate the corrosion resistance of Mg-based materials, which is because the self-healing ability can continuously repair the damaged areas of the coating, thus providing long-term corrosion protection. However, the self-healing coating cannot be effectively isolated in the contact between the surface and corrosive media.

Spontaneously, if a coating has both superhydrophobic and self-healing characteristics; that is, a self-healing superhydrophobic coating, which may be able to further improve the corrosion resistance of Mg alloys. It is reported, recently, that the self-healing superhydrophobic coating significantly enhances the corrosion resistance (usually by two to four orders of magnitude) and prolongs the service life of Mg alloys [102]. Two main methods are applied in the fabrication of self-healing superhydrophobic surfaces. The first one deals with storing hydrophobic components inside the rough nanomaterials [103]. In the case of damage to the surface, the hydrophobic components move to the damaged area and, hence, complete the self-healing process. The second approach has to do with the regeneration of micro-/nanoscale structures, which causes the damaged surfaces to regain their superhydrophobicity [104]. Unfortunately, low surface energy compounds for self-healing superhydrophobic coatings, such as perfluorinated compounds, organosilicon, and long alkyl chain compounds, may be harmful to the environment and expensive [105]. Besides, the self-healing superhydrophobic coating is still in its infancy. In the process of self-healing, the superhydrophobicity of the coating is difficult to be restored at the same time when the self-healing superhydrophobic coating is destroyed. Hence, it is urgent to devise a cheap, convenient, and environmentally friendly tactic to prepare self-healing superhydrophobic coatings on Mg alloys, and, thus, promote its industrial process.

## 5. Conclusions and Future Perspectives

Mg alloys, considered to be the best green material in the 21st century, have been employed in several industrial fields, yet the low corrosion resistance of Mg alloys limits their large-scale applications. Through surface modification, various compositions of films or coatings can be prepared to improve the corrosion resistance of Mg alloys and yield miscellaneous functional properties, including superhydrophobic and self-healing properties. This review summarizes the preparation methods and functional characteristics of these reported modification strategies for Mg alloys, and the following is a summary of our conclusions:

(1) Superhydrophobic coatings are projected as a suitable way to tackle the poor corrosion resistance of Mg alloys because the hydrophobic ability can usefully reduce the contact area between corrosive media and Mg alloys. However, the superhydrophobic coating is easily subjected to damage, and thus, loses superhydrophobicity. As a general rule, the prepared technology of superhydrophobic coatings on Mg alloys can be divided into one-step and two-step/multi-step methods. The two-step/multi-step method, such as micro-arc oxidation, chemical etching, and hydrothermal synthesis, first manufactures a rough structure on the Mg substrate, followed by modifying with the low surface-energy material; the one-step method, which consists of hydrothermal synthesis, electrodeposition, and sol-gel, is used to create a superhydrophobic substance that has both low surface energy and rough structure.
(2) Self-healing coatings are judged as a reasonable tactic that can meet the requirements of corrosion resistance of Mg alloys in some extreme service environments, which is because the self-healing property of the coating can entirely, or partially, repair the damaged area during applications, thereby extending the lifetime of the coating. Conceptually, these self-healing coatings can be compartmentalized into autonomous and non-autonomous coatings. Autonomous coatings can quickly respond to environmental or mechanical attacks related to corrosion or other destructive processes and release corrosion inhibitors or polymerizable healing agents. Non-autonomous self-healing coating can be truly repaired when exposed to external heat or light stimulation, which closes the damage and reforms the chemical or physical cross-linking inherent in the coating matrix.

In the future, a self-healing superhydrophobic coating is the development trend to improve the corrosion resistance of Mg alloys. For the superhydrophobic coating with self-healing properties, the active material is undoubtedly the core part of the coating; hence, novel and efficient active materials, such as supramolecules, are used properly to design the self-healing superhydrophobic coating. Besides, realizing the mutual coordination between multiple functions, mutual promotion is an important direction of functionally self-healing superhydrophobic coatings, which may be a promising modification strategy to endow the developed coating with robust superhydrophobicity and fast self-healing ability.

## Figures and Tables

**Table 1:** Synthesis of superhydrophobic coatings on Mg alloys by various preparation strategies.

References	Substrates	Synthesis Method	Wettability	Comments
[42]	AZ31B	Micro-arc oxidation	152°	Stearic acid-modified MAO surface provided the protracted corrosion guard of the AZ31 Mg alloy.
[70]	Mg-3.0Nd-0.2Zn-0.4Zr	Micro-arc oxidation	151°	The superhydrophobic film was successfully prepared by combining the MAO and the sol-gel method, which could effectively diminish the direct contact area between the sample and the aggressive medium.
[72]	AZ31	Chemical etching	154°	A simple method, involving etching with CuCl_2_, was used to fabricate a superhydrophobic surface on Mg alloys.
[52]	AZ31	Chemical etching	159°	Through a simple chemical etching (HCl aqueous solution) and surface modification (stearic acid-ethanol solution), a superhydrophobic coating with tunable water adhesion was prepared on the AZ31 alloy.
[33]	AZ91D	Hydrothermal synthesis	154°	A lotus seedpod bioinspired superhydrophobic surface was developed on the Mg alloy via an in situ hydrothermal synthesis technique, which rendered effective corrosion protection for the Mg substrate.
[73]	Mg-9Li	Hydrothermal synthesis	152°	One-step hydrothermal processing to develop the super-hydrophobic and corrosion-resistant coating on Mg-9Li alloy, using a mixed solution of stearic acid-ethanol-distilled.
[57]	Mg-2.5Y-1Ce-0.5 Mn	Hydrothermal synthesis	164°	Superhydrophobic coating of the main composition of CeO_2_ and Ce(CH_3_(CH_2_)_16_COO)_3_ with an allium giganteum-like structure shows excellent performance of anti-corrosion.
[74]	AZ31	Electrode-position	158°	an excellent anti-corrosion superhydrophobic DTMS coating was successfully fabricated on Mg alloy AZ31 by one-step electrodeposition in a relatively neutral solution.
[75]	AZ31B	Electrode-position	156°	The underlying LDH structure was prepared electrodeposition, which could form a passive-tion layer on the surface to protect the substrate as well as enhance the interface adhesion.
[7]	AZ31	Electrode-position	156°	A superhydrophobic coating with excellent corrosion resistance was successfully prepared on the AZ31 by one-step electrodeposition.
[68]	AZ31	Spraying	152°	PPS-PTFE/SiO_2_ superhydrophobic coatings with the fibrous-network structure were successfully fabricated by a simple spraying method, which has superior corrosion protection ability.
[76]	AZ31B	Spraying	157°	A robust MOF-organic compound superhydrophobic coating was created on the AZ31B alloy by a spraying method, which is a sustainable method of delaying metal corrosion.
[77]	MA8	Sol-gel	171°	A simple route combining laser processing with a sol-gel route was utilized for preparing a superhydrophobic coating, which could improve the anti-corrosion.

**Table 2:** Corrosion studies of functional coatings on Mg alloys; ^♣^ Superhydrophobic coating, ^♥^ self-healing coating, and ^♣♥^ Superhydrophobic and self-healing coating.

References	Substrate	Functional Coatings	Corrosion Media (3.5 wt.% NaCl)	*i*_corr_ (A·cm^−2^)		*E*_corr_ (V/SCE)		Inhibition Efficiency (*η*)	Immersion Time (h)
				Substrates	Coatings	Substrates	Coatings		
[59] ^♣^	AZ91D	Ni-Co/SA	-	1.5 × 10^−5^	9.2 × 10^−9^	−1.59	−0.172	0.9994	-
[96] ^♣^	AZ31	Ferric myristate	-	2.075 × 10^−5^	2.579 × 10^−8^	−1.167	−1.377	0.9988	72
[42] ^♣^	AZ31	MAO/SA	-	1.606 × 10^−4^	0.14 × 10^−7^	−1.524	−1.353	-	264
[97] ^♥^	AZ91D	Stannate/SA	-	1.104 × 10^−5^	1.132 × 10^−6^	−1.524	−0.803	0.3126	24
[56] ^♣^	AZ91D	Zn–Al LDHs/SA	-	8.8 × 10^−3^	2.6 × 10^−5^	−1.51	−1.22	0.9970	-
[72] ^♣^	AZ31	Etching with copper (II) chloride/SA	-	-	-	−1.59	−1.16	-	36
[98] ^♣,^^♥^	AZ31B	Cr (III) CCC/SA	-	6.09 × 10^−4^	0.643 × 10^−6^	−0.151	0.146	0.9989	-
[7] ^♣^	AZ31	Magnesium stearate	-	7.02 × 10^−5^	5.80 × 10^−8^	−1.542	−1.525	0.9992	168
[5] ^♣^	AZ31	PAPTMS/PP	-	4.96 × 10^−5^	9.08 × 10^−8^	−1.51	−1.38	0.9982	250
[76] ^♣^	AZ31B	GO/PPy/ZIF-8	-	7.779 × 10^−4^	1.133 × 10^−6^	−1.411	−1.509	0.9985	120
[54] ^♣^	AZ31	Mg(OH)_2_/PP	-	6.15 × 10^−5^	3.12 × 10^−9^	−1.46	−1.18	0.9999	250
[57] ^♣^	Mg-2.5Y-1Ce-0.5 Mn	CeO_2_@cerium stearate	-	4.22 × 10^−5^	8.06 × 10^−8^	−1.63	−1.16	0.9998	72

## Data Availability

Not applicable.
